# Factors Associated with Hypertension Among Men in Darkhan-Uul Province, Mongolia: A Cross-Sectional Study

**DOI:** 10.31372/20190404.1067

**Published:** 2020

**Authors:** Naoko Hikita, Megumi Haruna, Masayo Matsuzaki, Emi Sasagawa, Minoru Murata, Ariunaa Yura, Otgontogoo Oidovsuren

**Affiliations:** a Department of Midwifery and Women’s Health, Division of Health Sciences and Nursing, Graduate School of Medicine, The University of Tokyo, Japan; b Department of Children and Women’s Health, Division of Health Science, Graduate School of Medicine, Osaka University, Japan; c Mito Saiseikai General Hospital, Ibaraki, Japan; d Darkhan-Uul General Hospital, Darkhan-Uul, Mongolia; e Pharmaceutical Department, Mongolian National University of Medical Sciences, Darkhan-Uul Medical School, Mongolia

**Keywords:** noncommunicable disease, hypertension, cardiovascular disease, obesity, Mongolia

## Abstract

In Mongolia, cardiovascular disease is the leading cause of death, and prevalence of hypertension is very high. The aim of this study was to investigate the association between hypertension and sociodemographic factors and health-related behaviors among men in Darkhan-Uul Province, Mongolia. This cross-sectional study was conducted between November 2015 and January 2016. Men whose wives were pregnant with ≤ 20 weeks gestation and had attended antenatal health checkups at public health facilities were recruited in this study. The data were collected as part of a survey of pregnant women and their partners. Data were collected using self-administered questionnaires, anthropometry, and spot urine samples. A total of 224 men participated in the survey, and data from 209 participants were included in the analysis. Multiple logistic regression analysis showed that men with higher BMI had higher odds of hypertension than those with lower BMI (adjusted odds ratio [AOR]: 1.14, 95% CI: 1.03–1.26). Those with urinary cotinine > 100 ng/ml (smokers) had a lower risk of hypertension (AOR: 0.24, 95% CI: 0.09–0.67) compared to participants with urinary cotinine/ml (nonsmokers). This is the first study to investigate the association between hypertension and sociodemographic factors and health-related behaviors among men in Mongolia. Based on the findings of this study, clinicians responsible for public health in Mongolia should provide health education regarding the importance of weight control in preventing hypertension.

## Introduction

Noncommunicable diseases are the leading cause of death worldwide, and one of the global health challenges (World Health Organization, 2014, 2018). Among them, hypertension is a significant health problem because it increases the risk of coronary heart disease, chronic kidney disease, stroke, myocardial infarction, and heart failure (Collins et al., 1990; World Health Organization, 2007, 2013); thus, controlling blood pressure is crucial. However, two-thirds of the people with hypertension (639 million people) live in developing countries (Kearney et al., 2005), where there is less awareness of hypertension and therefore poor blood pressure control (Ibrahim & Damasceno, 2012).

The Mongolian STEPS survey reported that 30.5% of men and 24.5% of women among people aged 15–64 years had hypertension (Public Health Institute, 2013). Another survey in Mongolia reported that 12.7% of the participants were identified to have high risk for hypertension (Center for Health Development, 2017). Furthermore, another previous study reported that 17.4% of the participants from across Mongolia, had never heard the term “blood pressure,” and that this lack of knowledge was more common among younger participants and among men (Demaio et al., 2013).

It is well known that several factors contribute to hypertension, including high consumption of salty and fatty foods, low consumption of fruit and vegetables, aging, overweight and obesity, alcohol abuse, physical inactivity, psychological stress, and obstructive sleep apnea (Bradley & Floras, 2009; Brown et al., 2000; Doll, Paccaud, Bovet, Burnier, & Wietlisbach, 2002; Forman, Stampfer, & Curhan, 2009; Poirier et al., 2006; Stamler, Stamler, Riedlinger, Algera, & Roberts, 1978; Sun et al., 2010; Whelton, 1994; World Health Organization, 2014). In Mongolia, the prevalence of overweight or obesity was very high (49.1% of men and 59.9% of women were overweight or obese), and consumption of vegetable and fruits was less (Public Health Institute, 2013). Moreover, because outdoor temperatures are very low in winter (sometimes lower than –40 °C), physical inactivity might be a possibility. Although hypertension is a major health problem in Mongolia, no studies have evaluated the factors associated with hypertension. Identifying the factors associated with hypertension could help clinicians in imparting appropriate health education.

The aim of this study was to investigate the association between sociodemographic factors and health-related behaviors and hypertension among men in Darkhan-Uul Province in Mongolia.

## Methods

### Study Design and Population

This cross-sectional study was conducted in Darkhan-Uul Province, Mongolia. The participants were recruited from November 2015 to January 2016. Inclusion criteria of the recruitment were men whose wives were pregnant with ≤ 20 weeks gestation and had attended antenatal health checkups at public health facilities in Darkhan-Uul Province. Participants who could not understand Mongolian or who would have difficulty participating due to mental impairment were excluded from the study at the discretion of the healthcare staff. This study was conducted by the Mito Saiseikai General Hospital, as a baseline survey of the Japan International Cooperation Agency Partnership Program.

### Ethical Considerations

This study was approved by the Research Ethics Committee of the Graduate School of Medicine, the University of Tokyo, Japan (No. 10934), and the Ethical Review Board of the Ministry of Health, Mongolia (No. 06, November 19, 2015).

### Data Collection and Study Procedures

Participants completed a self-administered questionnaire, provided a spot urine sample, and had anthropometric measurements taken by study staff. The questionnaire recorded sociodemographic data, including age, level of education, and employment status; and lifestyle, including physical exercise within the last 30 days, alcohol consumption, and smoking status. Bodyweight and body fat were measured using a body composition analysis weight scale (BC-758, TANITA Corp., Tokyo, Japan). Height was determined by self-report, or by direct measurement if participants were unsure of their height. Body mass index (BMI; weight [kg]/height [m^2^]) was calculated using the bodyweight and height measures. After completing the questionnaire, participants’ blood pressure was measured twice while being seated, by the medical staff using a digital sphygmomanometer (ES-H55, TERUMO Corp., Tokyo, Japan). As people sometimes do not report their smoking status honestly, the spot urine sample was used to measure urinary cotinine, a biomarker for nicotine, in order to determine smoking status. Urinary cotinine was measured using an enzyme-linked immunosorbent assay (ELISA) (Calbiotech Inc., Spring Valley, CA, USA). Levels were categorized into three groups: < 5 ng/ml, 5–100 ng/ml, and > 100 ng/ml, based on the lower (5 ng/ml) and upper (100 ng/ml) limits of quantification for the test. These cut-off points were chosen because there is currently no accepted risk-free level of exposure to secondhand smoke (US Surgeon General, 2006) and in previous studies, urinary cotinine levels of nonsmokers did not exceed 100 ng/ml (Biber et al., 1987; Haufroid & Lison, 1998).

### Definition of Hypertension in this Study

For this study, participants were defined as hypertensive if they had two consecutive systolic blood pressure readings ≥ 140 mmHg, or two consecutive diastolic pressure readings ≥ 90 mmHg. Because it had previously been reported that 35.7% of Mongolian men had never had their blood pressure measured (Public Health Institute, 2013), participants’ self-reported history of hypertension was not considered in this study. In addition, participants were not asked about their use of antihypertensive medication; therefore, being on treatment for hypertension was not included in the definition of hypertension.

### Statistical Analysis

Distribution of the number of participants with and without hypertension was compared using the chi-square test, and Student’s *t*-test was used to compare the means for continuous variables. Variables with *p* values < .05 in bivariate analysis were selected as independent variables in a multiple logistic regression analysis. Age was also selected as an independent variable in the multiple logistic regression analysis because of its known association with hypertension. The Spearman rank correlation coefficient and Pearson’s correlation coefficient were used to evaluate multicollinearity in continuous variables, and Cramér’s measure of association was used in categorical variables. If the coefficient exceeded 0.7, one of the variables was removed from the multiple logistic regression model. IBM SPSS Statistics 25.0 for Windows (IBM Corp., Armonk, NY, USA) was used for analysis, and the level of significance was set at* p * < .05 (two-tailed).

## Results

### Participant Characteristics

A total of 227 sets of individual records were collected. Three of the participants completed the questionnaire twice, so their second set of measurements were excluded. In addition, three participants could not be matched to pregnant women, four participants had missing BMI data, and eight participants had missing blood pressure data. After these exclusions, the number of participants was reduced to 209.

Table 1 shows participant’s characteristics. The age (mean ± SD) was 28.3 ± 6.2 years, and of the 209 participants, 24 (11.5%) had hypertension. The prevalence of overweight and obesity was 42.6% (28.2% and 14.4%, respectively). The bivariate analysis found that bodyweight, BMI, BMI category, body fat, waist circumference, and urinary cotinine concentration had statistically significant associations with hypertension. Multicollinearity was found between bodyweight and BMI, BMI category, and waist circumference; between BMI and BMI category, and waist circumference; between waist circumference and BMI category, and the correlation coefficient exceeded 0.7. Thus, BMI was used as an independent variable in the multiple logistic regression analysis, and the other measures were excluded. Though the correlation coefficient between BMI and body fat was 0.615, since BMI connotes body fat, we excluded body fat from the multiple logistic regression analysis.

**Table 1:** Characteristics of Participants (n = 209)

	All		Normal		Hypertensiona		
	(*n* = 209)		(*n* = 185, 88.5%)		(*n* = 24, 11.5%)		
	Mean ± SD or *n* (%)		Mean ± SD or *n* (%)		Mean ± SD or *n* (%)		*p*^*b*^
Age (years)	28.3 ± 6.2						0.253
18–29	136	(65.1)	124	(91.2)	12	(8.8)	
30–39	62	(29.7)	52	(83.9)	10	(16.1)	
40–49	11	(5.2)	9	(81.8)	2	(18.2)	
Educational attainment							0.482
≤ Lower secondary school	27	(12.9)	23	(85.2)	4	(14.8)	
Upper secondary school	102	(48.8)	93	(91.2)	9	(8.8)	
≥ University	79	(37.8)	68	(86.1)	11	(13.9)	
Missing	1	(0.5)					
Employment status							0.423
Employed	108	(51.7)	95	(88.0)	13	(12.0)	
Self-employed	40	(19.1)	33	(82.5)	7	(17.5)	
Nomad	9	(4.3)	8	(88.9)	1	(11.1)	
Unemployed	49	(23.5)	46	(93.9)	3	(6.1)	
Missing	3	(1.4)					
Height (cm)	171.2 ± 7.0		170.9 ± 7.1		173.0 ± 6.6		0.166^c^
Bodyweight (kg)	73.4 ± 14.1		72.2 ± 13.3		82.3 ± 16.7		0.009^c^
Body mass index (kg/m^2^)	25.0 ± 4.1		24.7 ± 4.0		27.3 ± 4.3		0.003^c^
Underweight (≤18.5 kg/m^2^)	4	(1.9)	4	(100.0)	0	(0.0)	0.039
Normal (18.5–24.9 kg/m^2^)	116	(55.5)	106	(91.4)	10	(8.6)	
Overweight (25.0–29.9 kg/m^2^)	59	(28.2)	53	(89.8)	6	(10.2)	
Obese (≥30.0 kg/m^2^)	30	(14.4)	22	(73.3)	8	(26.7)	
Body fat (%)^d^	25.5 ± 8.3		25.1 ± 8.3		28.9 ± 8.4		0.036^c^
Waist circumference (cm)^e^	85.4 ± 12.2		84.5 ± 11.4		92.7 ± 15.7		0.023^c^
Physical exercise within last 30 days							0.937
Every day	46	(22.0)	40	(87.0)	6	(13.0)	
2–3 times/week	50	(23.9)	45	(90.0)	5	(10.0)	
2–3 times/month	40	(19.2)	35	(87.5)	5	(12.5)	
Not at all	65	(31.1)	58	(89.2)	7	(10.8)	
Prohibited	4	(1.9)	4	(100.0)	0	(0.0)	
Missing	4	(1.9)					
Alcohol consumption							0.317
Never	60	(28.7)	53	(88.3)	7	(11.7)	
Monthly or less	97	(46.4)	89	(91.8)	8	(8.2)	
2–4 times a month	49	(23.4)	40	(2.0)	9	(18.4)	
2–3 times a week	2	(1.0)	2	(100.0)	0	(0.0)	
Missing	1	(0.5)	1	(100.0)	0	(0.0)	
Smoking status							0.331
Daily smoker	92	(44.0)	85	(92.4)	7	(7.6)	
Occasional smoker	34	(16.3)	28	(82.4)	6	(17.6)	
Nonsmoker	80	(38.3)	69	(86.3)	11	(13.7)	
Don’t know	3	(1.4)	3	(100.0)	0	(0.0)	
Urinary cotinine concentration							0.023
<5 ng/ml	44	(21.0)	34	(77.3)	10	(22.7)	
5–100 ng/ml	30	(14.4)	26	(86.7)	4	(13.3)	
>100 ng/ml	133	(63.6)	123	(92.5)	10	(7.5)	
Missing	2	(1.0)					

SD: standard deviation.

^a^Hypertension was defined if participants had twice consecutive systolic blood pressure readings ≥140 mmHg, or twice consecutive diastolic pressure readings ≥90 mmHg.

^b^Chi-squared test.

^c^Student’s *t*-test.

^d^Missing for 14 participants.

^e^Missing for 3 participants.

### Factors Associated with Hypertension

Table 2 shows the results of the multiple logistic regression analysis of factors associated with hypertension. Participants with higher BMI had higher odds of hypertension than those with lower BMI (adjusted odds ratio [AOR] = 1.14, 95% CI: 1.03–1.26). Furthermore, compared to men whose urinary cotinine concentration levels were < 5 ng/ml, those with > 100 ng/ml had lower odds of hypertension (AOR = 0.24, 95% CI: 0.09–0.67). Significant differences were not observed among age groups.

**Table 2:** Factors associated with hypertension

	Crude odds ratio	95% CI^†^	*p* value	Adjusted odds ratio	95% CI^†^	*p* value
Age						
18–29	Reference			Reference		
30–39	1.99	(0.81–4.89)	0.135	1.89	(0.72–4.97)	0.196
40–49	2.30	(0.44–11.87)	0.321	1.44	(0.25–8.37)	0.682
Body mass index	1.15	(1.04–1.27)	0.005	1.14	(1.03–1.26)	0.014
Urinary cotinine concentration						
< 5 ng/ml	Reference			Reference		
5–100 ng/ml	0.52	(0.15–1.86)	0.316	0.45	(0.12–1.68)	0.234
>100 ng/ml	0.28	(0.11–0.72)	0.008	0.24	(0.09–0.67)	0.006

Multiple logistic regression analysis adjusted for the variables in this table.

Hypertension was defined if participants had twice consecutive systolic blood pressure readings ≥140 mmHg, or twice consecutive diastolic pressure readings ≥90 mmHg.

^†^: CI = confidence interval

## Discussion

This is the first study to investigate the association between sociodemographic factors and health-related behaviors and hypertension among men in Mongolia. We found that participants with higher BMI had higher odds of hypertension than those with lower BMI, and participants with urinary cotinine > 100 ng/ml had a lower risk of hypertension compared to those with urinary cotinine < 5 ng/ml.

The prevalence of hypertension in this study (11.5%) was lower than that found in the STEPS survey (30.5%), which targeted men aged 15–64 years (Public Health Institute, 2013), and the government reports (12.7%) (Center for Health Development, 2017). This may have been because the participants in this study were younger on an average. As hypertension tends to become more prevalent with age, it is beneficial to manage factors related to lifestyle earlier in life when it may be comparatively easier to adopt changes in lifestyle. Therefore, studying factors associated with hypertension among relatively younger men could provide useful information to the clinicians in Mongolia regarding the type of health education they need to impart, in order to prevent hypertension in younger men.

The prevalence of overweight and obesity among men in this study was 42.6%, which is similar to that found in the STEPS survey (49.0%) (Public Health Institute, 2013), and another Ministry of Health survey (48.8%) (Ministry of Health, National Center for Public Health, United Nations Children’s Fund, 2017). In Mongolia, the prevalence of overweight and obesity was high even among younger men, thus putting them at risk of developing health problems in the future. As it has been reported that obesity is a major risk factor of hypertension (Doll et al., 2002; Sun et al., 2010), health education should include messages on the importance of weight control.

### Factors Associated with Hypertension

In this study, participants with higher BMI had higher odds of hypertension than those with lower BMI. This result confirmed the results of previous studies (Brown et al., 2000; Stamler et al., 1978). As it has been reported that almost half of the adults in Mongolia are overweight or obese (Ministry of Health, National Center for Public Health & United Nations Children’s Fund, 2017), focusing on measures against obesity could prevent hypertension.

This study revealed that participants with urinary cotinine levels > 100 ng/ml had a lower risk of hypertension than those with urinary cotinine levels < 5 ng/ml. The reasons for this finding are unclear. Although smoking is well known to cause a short-term increase in blood pressure (Failla et al., 1997; Giannattasio et al., 1994; Niedermaier et al., 1993), some previous studies have shown that smoking reduces systolic blood pressure (Alomari & Al-Sheyab, 2016; Kim, Han, Kang, Kim, & Kang, 2017); however, this result should be interpreted with caution. Though the mechanism of how smoking affects blood pressure is still unclear, some studies have inferred that nicotine induces angiogenesis (Heeschen et al., 2001), or a mediator such as enkephalin-like peptides might have a vasodilatory effect (Hexum & Russett, 1987). Nevertheless, smoking is one of the major risk factors for cardiovascular disease and cessation of smoking has a beneficial impact on cardiovascular disease (National Center for Chronic Disease Prevention and Health Promotion Office on Smoking and Health, 2014; World Health Organization, 2007); hence, we would not advocate smoking as a way of preventing hypertension, based on the results of this study.

### Strengths and Limitations

This is the first study to investigate the association between hypertension and sociodemographic status and health-related behavior among men in Mongolia. However, it has several limitations. First, these results cannot be generalized for the general population of Mongolian men, as the study participants were partners of pregnant women, and were not representative of the male population of Mongolia; moreover, the sample size was small. Second, the definition of hypertension in this study was not based on a clinical diagnosis. However, we measured participants’ blood pressure twice and defined hypertension based on systolic and diastolic blood pressure; therefore, we consider our classification to be relatively reliable, although some participants who were taking antihypertensive medication might have been classified as normotensive. Third, because we used self-reported height to calculate the BMI, and participants may not have reported their height accurately, BMI calculations of some participants may have been inaccurate. Finally, although urinary cotinine is a reliable indicator of smoking status, we were unable to distinguish occasional smokers from heavy smokers.

## Conclusions

This is the first study to investigate the association between hypertension and sociodemographic factors and health-related behaviors among men in Mongolia. This study revealed that participants with higher BMI had a higher risk of hypertension, and participants with urinary cotinine levels > 100 ng/ml had a lower risk of hypertension. As hypertension increases the risk of cardiovascular diseases such as stroke, myocardial infarction, and heart failure, clinicians should educate patients regarding the importance of weight control. Further research that covers a broad spectrum of the Mongolian population, and long-term population-based cohort studies, should be conducted to gain additional information on risk factors for hypertension among men in Mongolia.

## Acknowledgments

We thank Battsengel Buyannemekh at the Mongolian Health Agency, and Erdenetulga Buyandalai at Darkhan-Uul General Hospital for coordinating the research.

## Declaration of Conflicting Interests

The authors declared no potential conflicts of interest with respect to the research, authorship, and/or publication of this article.

## Funding

This study was funded by the Japan International Cooperation Agency (JICA) Partnership Program and the Research Assistant Program from the Graduate School of Medicine at the University of Tokyo.
